# Increased lignocellulosic inhibitor tolerance of *Saccharomyces cerevisiae* cell populations in early stationary phase

**DOI:** 10.1186/s13068-017-0794-0

**Published:** 2017-05-04

**Authors:** Venkatachalam Narayanan, Jenny Schelin, Marie Gorwa-Grauslund, Ed WJ van Niel, Magnus Carlquist

**Affiliations:** Division of Applied Microbiology, Department of Chemistry, Lund University, P.O. Box 124, SE 221 00 Lund, Sweden

**Keywords:** Carbon starvation, Stress tolerance, Acetic acid, Vanillin, Furfural, Reactive oxygen species, Intracellular pH, Quiescence, Population heterogeneity

## Abstract

**Background:**

Production of second-generation bioethanol and other bulk chemicals by yeast fermentation requires cells that tolerate inhibitory lignocellulosic compounds at low pH. *Saccharomyces cerevisiae* displays high plasticity with regard to inhibitor tolerance, and adaptation of cell populations to process conditions is essential for reaching efficient and robust fermentations.

**Results:**

In this study, we assessed responses of isogenic yeast cell populations in different physiological states to combinations of acetic acid, vanillin and furfural at low pH. We found that cells in early stationary phase (ESP) exhibited significantly increased tolerance compared to cells in logarithmic phase, and had a similar ability to initiate growth in the presence of inhibitors as pre-adapted cells. The ESP cultures consisted of subpopulations with different buoyant cell densities which were isolated with flotation and analysed separately. These so-called quiescent (Q) and non-quiescent (NQ) cells were found to possess similar abilities to initiate growth in the presence of lignocellulosic inhibitors at pH 3.7, and had similar viabilities under static conditions. Therefore, differentiation into Q-cells was not the cause for increased tolerance of ESP cultures. Flow cytometry analysis of cell viability, intracellular pH and reactive oxygen species levels revealed that tolerant cell populations had a characteristic response upon inhibitor perturbations. Growth in the presence of a combination of inhibitors at low pH correlated with pre-cultures having a high frequency of cells with low pH_i_ and low ROS levels. Furthermore, only a subpopulation of ESP cultures was able to tolerate lignocellulosic inhibitors at low pH, while pre-adapted cell populations displayed an almost uniform high tolerance to the adverse condition. This was in stark contrast to cell populations growing exponentially in non-inhibitory medium that were uniformly sensitive to the inhibitors at low pH.

**Conclusions:**

ESP cultures of *S. cerevisiae* were found to have high tolerance to lignocellulosic inhibitors at low pH, and were able to initiate growth to the same degree as cells that were pre-adapted to inhibitors at a slightly acidic pH. Carbon starvation may thus be a potential strategy to prepare cell populations for adjacent stressful environments which may be beneficial from a process perspective for fermentation of non-detoxified lignocellulosic substrates at low pH. Furthermore, flow cytometry analysis of pH_i_ and ROS level distributions in ESP cultures revealed responses that were characteristic for populations with high tolerance to lignocellulosic inhibitors. Measurement of population distribution responses as described herein may be applied to predict the outcome of environmental perturbations and thus can function as feedback for process control of yeast fitness during lignocellulosic fermentation.

**Electronic supplementary material:**

The online version of this article (doi:10.1186/s13068-017-0794-0) contains supplementary material, which is available to authorized users.

## Background

Adverse impacts of climatic change and concerns over energy security could be abated by replacing petrochemicals with chemicals produced from lignocellulose, which is the most abundant renewable feedstock on the planet and is available from industrial and agricultural residues. Intense research and development for decades have led to the onset of commercial scale production of lignocellulosic bioethanol using the industrial workhorse *Saccharomyces cerevisiae*. Although many improvements could be done in biomass pretreatment [1, 2], enzymatic hydrolysis [3] and inhibitor detoxification [4], the robustness of *S. cerevisiae* towards adverse process conditions is still a key engineering target to increase productivity, avoid loss of fermentable sugars and therefore reduce production costs [5]. An important hurdle to overcome for maintaining high cell activity is the negative effect of lignocellulosic inhibitors produced by the most common pretreatment methods; these include furaldehydes such as furfural and hydroxymethylfurfural (HMF), phenolics such as vanillin and 4-hydroxybenzoic acid and weak organic acids such as acetic acid, formic acid and levulinic acid (see reviews [5–7]).

Cell tolerance to lignocellulosic inhibitors is a highly plastic phenotype and depends on the environment that the cell population has experienced before exposure. For example, pre-cultivation in lignocellulosic hydrolysate containing furfural and HMF leads to induced expression of genes coding for specific NADPH-dependent oxidoreductases, e.g. Adh6 [8], that reduce the aldehyde moiety into less inhibitory furfuryl alcohols resulting in a shortened latency phase in the fermentation [9]. Tolerance to vanillin is similarly correlated to increased reduction to the less toxic vanillyl alcohol [10]. Also the tolerance to acetic acid at low pH is increased by pre-cultivation in medium supplemented with acetic acid at slightly acidic pH [11]. The acid tolerance is partly caused by an induced expression of the *HAA1* gene coding for a global transcription factor that activates multiple genes, including *TPO1* and *TPO2* coding for drug/H+-antiporters which export dissociated acetate from the cytoplasm [12, 13]. For these reasons, improved fermentation of lignocellulosic substrates can be reached through adapting cell populations by pre-exposure to moderate inhibitor levels in the pre-cultivation step [14, 15].

The level of cellular resistance to a specific stress is determined both by stress-specific and general mechanisms. For example, it was previously found that a slow growth rate correlates with increased tolerance towards a number of seemingly non-related stresses [16]. The extreme case are cells in stationary phase (SP), which are characterized by increased cell robustness to heat shock, osmotic stress, freeze–thaw stress and weak acid stress [17–21]. The higher robustness of SP-cells is often explained by activation of multiple cellular regulatory events upon nutrient starvation, including the environmental stress response (ESR), which leads to adjustment of cellular resources to promote survival in adjacent environments (see reviews [22, 23]). Based on this, it can be proposed that increased tolerance to lignocellulosic conditions may be reached without pre-exposure to inhibitors, for example, by allowing cells to reach SP by carbon starvation prior to the fermentation step.

The aim of the current study was to investigate correlations between the physiological state of yeast populations and their aptitude to tolerate combinations of lignocellulosic inhibitors (vanillin, furfuraldehyde and acetic acid) at low pH. In particular, physiological responses of cells in SP, including the previously described quiescent (Q) and non-quiescent (NQ) cells [24–26], were investigated in detail. Furthermore, flow cytometry (FCM) measurements of cell viability, the intracellular pH (pH_i_) and reactive oxygen species (ROS) levels were applied to compare the responses of cell populations in early stationary phase (ESP) to those of cells in logarithmic phase (LP), and cells pre-adapted to lignocellulosic inhibitors.

## Methods

### Strains and media

*Saccharomyces cerevisiae* strains used in this study are listed in Table 1. They were stored at −80 °C in Yeast Peptone (YP) medium containing 10 g L^−1^ yeast extract, 20 g L^−1^ peptone and 20 g L^−1^ glucose supplemented with 30% (v/v) glycerol and maintained on YP agar plates containing 10 g L^−1^ yeast extract, 20 g L^−1^ peptone, 20 g L^−1^ glucose and 15 g L^−1^ agar. A chemically defined medium [27] with 20 g L^−1^ glucose, buffered to pH 3.7, 5.0 or 6.5 with 50 mM potassium hydrogen phthalate [28] and supplemented with or without 6 g L^−1^ acetic acid, 0.75 g L^−1^ furfural and 0.2 g L^−1^ vanillin was used in all aerobic growth experiments. Vitamins, trace elements, furfural and vanillin were filter-sterilized to avoid changes in composition due to evaporation during autoclavation. *Escherichia coli* strain NEB5α (New England Biolabs) was recovered from 25% glycerol stock stored at −80 °C and used for subcloning of plasmid DNA and further propagation. Luria–Bertani broth (LB) (5 g L^−1^ yeast extract, 10 g L^−1^ tryptone and 5 g L^−1^ NaCl, pH 7.5) medium was used for culturing *E. coli* and 50 mg L^−1^ ampicillin was added to the LB medium when required. Media components were purchased from Sigma-Aldrich (Sweden), unless mentioned otherwise.

**Table 1:** *S. cerevisiae* strains used in this study

Strain	Genotype	Source
CEN.PK 113-7D	MATa MAL2-8c SUC2	EUROSCARF, Frankfurt, Germany
CEN.PK 113-5D	MATa MAL2-8c SUC2 *ura3*	EUROSCARF, Frankfurt, Germany
TMB3800	CEN.PK 113-5D; pYES-*P*_*ACT1*_-pHlourin (URA3)	This study
TMB3500	Wild brewer’s strain	[15]

### Construction of *S. cerevisiae* strain expressing pHluorin

Competent *E. coli* NEB5α cells were prepared using the RbCl method described in the subcloning notebook from Promega, which is adapted from the method described by [29]. The competent cells were transformed according to the supplier’s instructions (New England Biolabs). Bacterial transformants were selected on solid LB plates (15 g L^−1^ agar), supplemented with ampicillin (50 mg L^−1^), for 16 h at 37 °C. Plasmid preparation from *E. coli* transformants was performed using GeneJet™ Plasmid Miniprep kit (Thermo Scientific, Waltham, USA). *S. cerevisiae* CEN.PK 113-5D was grown in liquid YPD medium for 14–16 h at 30 °C and 180 rpm in a rotary shake incubator (New Brunswick, Enfield, CT, USA) when preparing the strain for transformation. It was transformed with the URA3-based 2µ episomal plasmid pYES-pACT1-pHluorin [30] using the high-efficiency LiAc method [31], and the engineered yeast strain (henceforth mentioned as TMB3800) was selected on YNB-glucose plates (6.7 g L^−1^ Yeast Nitrogen Base without amino acids (Becton, Dickinson and Company, USA) supplemented with 20 g L^−1^ glucose and 15 g L^−1^ agar).

### Aerobic batch cultivation in shake flasks

Cultures were grown in a rotary shake incubator at 180 rpm at 30 °C and cell concentrations were determined by optical density (OD) at 620 nm (Spectrophotometer U-1800, Hitachi, Berkshire, UK). Seed cultures were cultivated from single colonies of *S. cerevisiae* strains (from solid media) in 5 mL defined medium at pH 6.5 in a 50-mL conical tube until late exponential phase. Cells from the seed culture were harvested by centrifugation at 3056*g* for 5 min at 4 °C (Eppendorf centrifuge 5810 R, USA), washed with saline solution (0.9% NaCl) and subsequently used for inoculation of the pre-culture at an initial OD_620_ of 0.5. Cells from the pre-culture were harvested at different phases of cultivation and used to inoculate the final cultivation. Pre-cultivation and subsequent cultivations were performed in baffled shake flasks with the medium volume equivalent to 10% of baffled shake volume to maintain adequate aeration. All the experiments were carried out in biological replicates (*n* = 2 or 3) and measurements were carried out in technical triplicate.

### Flotation

TMB3500 was grown for 24 h until ESP, subsequently Q- and NQ-cells were separated by flotation [32]. For the flotation procedure, three different sterile colloidal solutions of non-toxic silanized silica particles in 0.9% NaCl, all formulated by QRAB (Alunda, Sweden) and produced by FertiPro N.V., Beernem, Belgium were used. The densities of the solutions were adjusted to previously reported buoyant cell densities for Q- (1.14 g mL^−1^) and NQ-cells (1.10 g mL^−1^) [24] as follows: BactXtractor-L (BX-L; density, 1.06 g mL^−1^), BactXtractor-M (BX-M; density, 1.12 g mL^−1^) and BactXtractor-H (BX-H; density, 1.29 g mL^−1^). The final densities of BX-L and BX-M were reached after dilution of BX-H with sterile 0.9% NaCl and measured using a DMA46 density metre (Instrument AB Lambda, Stockholm, Sweden). The flotation media were stored at 4 °C. Initially cells from an ESP culture of TMB3500 were harvested by centrifugation and homogenously re-suspended in 3 mL BX-H in a 15-mL Falcon Tube (Sarstedt, Nümbrecht, Germany). A discontinuous gradient was then created by the careful addition of 6 mL of BX-M followed by 2 mL of BX-L. The tube was centrifuged at 3056 g for 60 min at 4 °C using a swing-out centrifuge (Sigma 4-15C, Qiagen, Sweden) to separate Q- and NQ-cells. The two resulting cell fractions, due to differences in buoyant densities (Q-cells at the lower interphase between BX-H and BX-M layers, *δ* > 1.12; NQ-cells at the upper interphase between BX-M and BX-L layers, *δ* > 1.06), were collected using a syringe and needle. Each cell fraction was subsequently washed with sterile 0.9% NaCl and centrifuged at 3056*g* for 5 min at 4 °C. Cells from each fraction were visualized in the microscope (Nikon optiphot with Zeiss axiscam MRm, Sweden) and were characterized further in subsequent growth analyses.

### Analysis of responses of unsorted ESP-, Q- and NQ-cells to lignocellulosic inhibitors

TMB3500 was pre-cultured for 24 h to reach ESP and cells were harvested by centrifugation at 3056 g for 5 min at 4 °C. Unsorted ESP-cells, Q-cells and NQ-cells were inoculated at an OD_620_ of 0.5 in 200 μL of 15 different media (Table 2) with varied concentrations of inhibitors (furfural, vanillin, acetic acid) at pH 3.7 in 96-well microtiter plates covered with a transparent plastic film (Breathe easy, Diversified biotech, USA) to prevent evaporation. Concentrations of the inhibitors were defined with a circumscribed central composite design equation (*ccdesign*) using Matlab (Release R2015a, The MathWorks, Inc., Natick, MA, USA). Growth was followed for 40 h by measuring OD_620_ with a multiscan ascent spectrophotometer (ThermoFisher Scientific, Sweden). Three-way ANOVAs (*anovan*) using Matlab were performed to see any effect of individual inhibitors on growth after 14 h with the different inocula (Unsorted ESP-, Q- and NQ-cells) (Additional file 1: Tables S1–3). A principle component analysis (PCA) of the whole dataset with ESP-, Q- and NQ-cells as loads and medium (M1-15) as scores was performed using the *pca* function in Matlab. Lag times and maximum specific growth rates were calculated by fitting the raw data to the modified Gompertz growth equation [33] using the Solver function to minimize sum of least squares in Excel (Microsoft, 2013, USA).

**Table 2:** Inhibitor compositions of the media as defined by circumscribed central composite design

Media	Acetic acid (g L^−1^)	Furfural (g L^−1^)	Vanillin (g L^−1^)
1	1.00	0.50	0.50
2	1.00	0.50	1.50
3	1.00	1.50	0.50
4	1.00	1.50	1.50
5	6.00	0.50	0.50
6	6.00	0.50	1.50
7	6.00	1.50	0.50
8	6.00	1.50	1.50
9	0.00	1.00	1.00
10	7.70	1.00	1.00
11	3.50	0.16	1.00
12	3.50	1.84	1.00
13	3.50	1.00	0.16
14	3.50	1.00	1.84
15^a^	3.50	1.00	1.00

^a^ Medium 15 was used for 9 technical replicates

### Flow cytometry analysis

A BD Accuri™ C6 flow cytometer equipped with a Csampler (Becton–Dickinson, NJ, USA) was used to measure viability [34] and ROS [35], as described previously. Quality control of the instrument was made with 6 and 8 peak fluorescent calibration beads. The fluidics was set to medium flow rate (35 µL s^−1^), the threshold was set to 50,000 on the forward scatter channel, and 20,000 or 100,000 cells were collected at a rate between 3000 and 6000 events s^−1^. For determination of cell viability, 1 × 10^7^ cells mL^−1^ in phosphate-buffered saline solution (PBS) (8 g L^−1^ NaCl, 0.2 g L^−1^ KCl, 1.42 g L^−1^ Na_2_HPO_4_ and 0.24 g L^−1^ KH_2_PO_4_, pH 7.4) were stained with propidium iodide (PI) (1 µg mL^−1^) and incubated in the dark for 10 min. A blue laser (488 nm) was used for the excitation, and PI emission was collected at 585/40 nm. For determination of ROS, 1 × 10^6^ cells mL^−1^ in PBS solution were stained with dihydroethidium (DHE) (50 µg mL^−1^) and incubated in the dark for 20 min. DHE permeates into cells and gets oxidized to ethidium when exposed to superoxide in a dose-dependent manner. Ethidium then intercalates with DNA and emits red fluorescence proportional to intracellular ROS [35, 36]. A blue laser (488 nm) was used for the excitation, and DHE emission was collected at 585/40 nm. Cells in logarithmic phase grown in defined medium were used as live control and cells treated with 70% ethanol for 20 min were used as positive control for analysis of both viability and ROS. Autofluorescence was measured for unstained exponentially growing cells (CEN.PK 113-7D).

A Moflo XPD cell sorter (Becton–Dickinson, NJ, USA) with physically separated laser lines was used for ratiometric flow cytometry analysis of pHluorin fluorescence, as described previously [37]. Calibration of the instrument was made with fluorescent calibration beads (SPHERO Ultra rainbow fluorescent particles, 3.01 μM, Spherotech, USA). The threshold was set based on forward scatter obtained from blue laser (488 nm), and 100,000 cells were collected at a rate of approximately 5000 events/s. Excitation of pHluorin was made with a blue laser (488 nm) and a violet laser (405 nm), and the corresponding fluorescence emissions were collected with bandpass filters at 529/28 nm and 542/50 nm. The pH dependence of pHluorin was confirmed by measuring the ratio of fluorescence from the different excitation wavelengths, R405/488, in permeabilized cells in sodium phosphate buffer (0.2 M) at different pH ranging from 5.7 to 8. Permeabilization was made by incubating cells in PBS buffer (50 mM, pH 6.5) supplemented with digitonin (0.04 mM) for 15 min at room temperature on a turning table, as described previously [30].

Flow cytometry standard (FCS) data files were exported from the BD Accuri C6 software (BD Biosciences, USA) or saved directly from the Kaluza software (Beckman coulter, USA) and analysed with FlowJo v10.1 (FlowJo, LLC Ashland, OR, USA). For determination of percent viable cells, the gate was defined based on PI fluorescence (585/40 nm) of live and dead cell controls samples. The live control sample was exponentially growing cells in defined mineral medium, and the dead control was prepared by incubating cells in 70% ethanol for at least 30 min. An initial noise signal reduction was made for all samples by gating single cells based on the height and area of the forward scatter signal. pHluorin population was gated from autofluorescence (measured with a CEN.PK 113-7D strain without expression of pHluorin) using a bivariate plot of emissions at 542/50 nm and 529/28 nm, and excitation with the violet laser (405 nm) and the blue laser (488 nm), respectively. The pHluorin excitation ratio, R (F405/F488), for each cell was subsequently calculated using the derived parameter function in Flowjo. R (F405/488) for exponentially growing cells was used to differentiate cells with low and high pH. The cut-off was defined as the value (*R* = 0.400) dividing the population to two fractions: 1% lowest percentile (low pHi) and 99% highest percentile (high pHi) of exponentially growing cells. High and low ROS levels were defined similarly as the fluorescence intensity (height) at 585/50 nm dividing the LP-cell population in 1% lowest percentile (low ROS) and 99% highest percentiles (high ROS).

## Results

### Tolerance to acetic acid is growth phase dependent

**Fig. 1:**
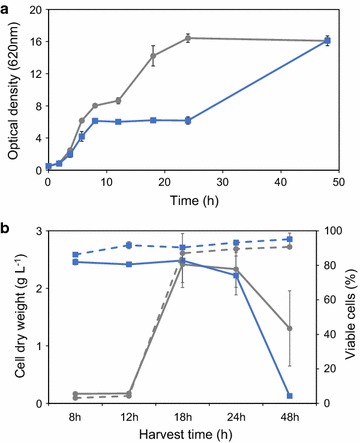
Yeast response to acetic acid and low pH. **a** Pre-cultivation of the *S. cerevisiae* TMB3500 strain in aerobic batch mode in defined mineral medium at pH 5.0 without (*round*) or with 6 g L^−1^ acetic acid (*square*). **b** Cell dry weight (*full lines*) and % viability (*dotted lines*) after 24 h aerobic batch cultivation in defined mineral medium at pH 3.7 with 6 g L^−1^ acetic acid from pre-cultures at pH 5.0 with (*blue*) and without (*grey*) 6 g L^−1^ acetic acid. *Error bars* represent standard error of biological duplicates (*n* = 2)

Acetic acid is one of the most important adversaries during fermentation of lignocellulosic substrates. Yeast cells display relatively high tolerance to acetic acid at low pH; however, the level of tolerance depends on several mechanisms that are differently regulated depending on additional extracellular conditions. To shed light on to whether the acetic acid tolerance phenotype can be induced by carbon starvation instead of pre-cultivation in the presence of acetic acid, cells were cultivated in aerobic batch mode in a defined mineral medium and harvested at different growth phases, i.e. at log phase (LP) (8–12 h), early stationary phase (ESP) (18–24 h) and late stationary phase (48 h). In parallel, cells were also pre-cultivated in a medium with 6 g L^−1^ acetic acid at pH 5.0, since this condition was previously shown to induce the desired acid tolerance [11]. Supplementation of acetic acid in the pre-cultivation medium inhibited growth already at pH 5.0, as reflected by an extended diauxic phase as well as a reduced growth rate during glucose assimilation compared to the reference cultivation (Fig. 1a). With a pKa for acetic acid of 4.76, the concentration of undissociated acetic acid experienced by the cells at pH 5.0 was approximately 2.2 g L^−1^. Although growth profiles differed substantially, the final biomass concentrations in the two pre-cultivations were the same with or without acetic acid in the medium. The cells of each of the pre-cultivations were re-inoculated in a new medium supplemented with 6 g L^−1^ acetic acid at pH 3.7.

The LP-cells not pre-exposed to acetic acid did not proliferate in the new medium (Fig. 1b). The poor tolerance to acetic acid at low pH was also clear from the poor viability after 24 h in the new medium (3–4% of the population). In contrast, pre-adapted cells that were pre-cultured the same length of time (8–12 h) were able to generate a cell biomass of ca 2.5 ± 0.1 gdw L^−1^ and cell viability was around 90% after 24 h (Fig. 1b).

The ESP-cells were able to initiate proliferation in the new medium, whether or not they were pre-grown in the presence of acetic acid at pH 5.0, resulting in a high biomass titre (2.4 gdw L^−1^) and viability (87%) after 24 h. This demonstrates that ESP-cells indeed had an induced acetic acid tolerance. In late SP (48 h pre-cultivation), cells displayed a reduced ability to grow, thus demonstrating a lower tolerance to acetic acid at low pH. However, for this long pre-cultivation time, the biomass titre was substantially lower if cells were pre-adapted than not (1.30 ± 0.65 gdw L^−1^ vs. 0.13 ± 0.01 gdw L^−1^). Survival was not correlated to growth for ESP- and late SP-cells, as shown by a high viability (>90%) even under static conditions (Fig. 1b).

### Tolerance of ESP-cell subpopulations to combinations of lignocellulosic inhibitors

**Fig. 2:**
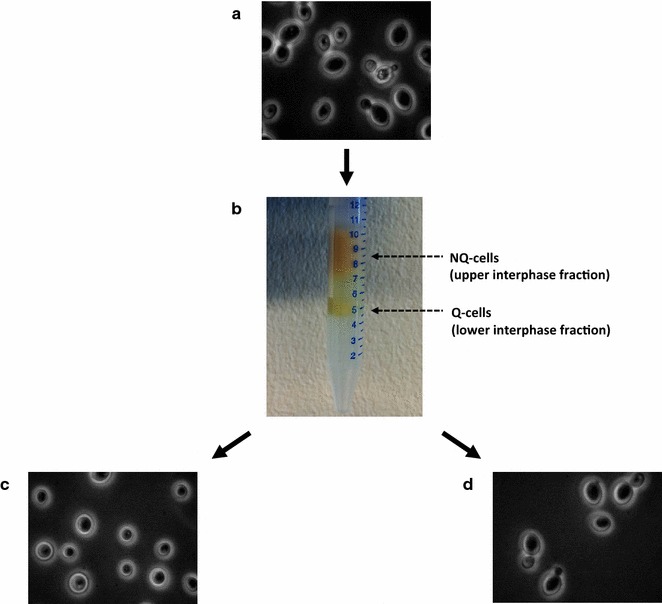
Separation of *S. cerevisiae* TMB3500 in ESP using flotation. **a** Unsorted SP-cells. **b** Discontinuous density gradient after centrifugation.** c** Q-cells** d** NQ-cells

The conditional boundaries of ESP-cells to tolerate lignocellulosic inhibitors at pH 3.7 were further mapped by performing a series of cultivations in microtiter plates in media supplemented with different levels of acetic acid, vanillin and furfural according to a 2^3^ factorial design (Table 2). In addition, we reasoned that the observed increase in tolerance to acetic acid of ESP-cells was due to their differentiation into Q-cells, which were previously shown to possess increased tolerance to heat shock and oxidative stress compared to NQ-cells [24]. To investigate this further, Q- and NQ-cell subpopulations were separated and both were analysed in parallel with the unsorted ESP-cells. Indeed, two cell fractions with different buoyant densities were separated using flotation (Fig. 2), whereas cells in exponential phase cells could not, which is in accordance with the previous studies [24, 25]. When analysed under the microscope, the Q-cells were round without buds in contrast to the NQ-cells that were a heterogeneous mixture of budding and non-budding cells (Fig. 2).

**Fig. 3:**
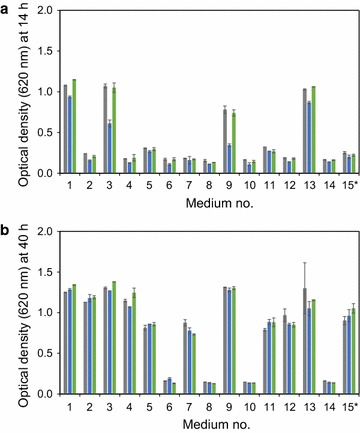
Cell density after (a) 14 h and (b) 40 h cultivation in media M1-M15. Unsorted SP-cells (*grey*), Q-cells (*blue*) or NQ-cells (*green*) as inoculum. *Error bars* represent standard deviation of the mean for biological duplicates (*n* = 2) except for *M15 (*n* = 18)

The three cell populations (ESP-cells, Q-cells and NQ-cells) were each re-inoculated in 15 different medium compositions (M1-M15) and cell growth was monitored for 40 h. Concentration of glucose was low (20 g L^−1^) to avert osmotic stress to the yeast cell and to keep ethanol levels low, thereby removing their inhibitory effect as significant factors on the growth of the populations. Growth was observed in all media except M6, M8, M10 and M14, although the final biomass generated was substantially different (Fig. 3). To distinguish the individual and combinatory effect of inhibitors on growth, a 3-way analysis of variation (ANOVA) was used separately on each dataset generated for each test (Additional file 1: Tables S1–3). The first measured time point (14 h) (Fig. 3a) was used as input for the ANOVA, since the largest effect of these different inocula was observed in the latency phase. From the ANOVAs, it was seen that acetic acid, furfural and vanillin inhibited growth significantly (*p* < 0.001) for all inocula, although the effect of furfural was the smallest. The synergistic effect of two inhibitors (acetic acid and vanillin, acetic acid and furfural or vanillin and furfural) on growth was significant in all cases when using Q-cells as inoculum, indicating that this cell subpopulation required a longer latency period under the applied conditions (Additional file 1: Table S2). However, for unsorted ESP-cells and NQ-cells, a combinatorial effect was significant only for acetic acid and vanillin, and none of the combinations with furfural (*p* > 0.01) (Additional file 1: Tables S1 and S3).

**Fig. 4:**
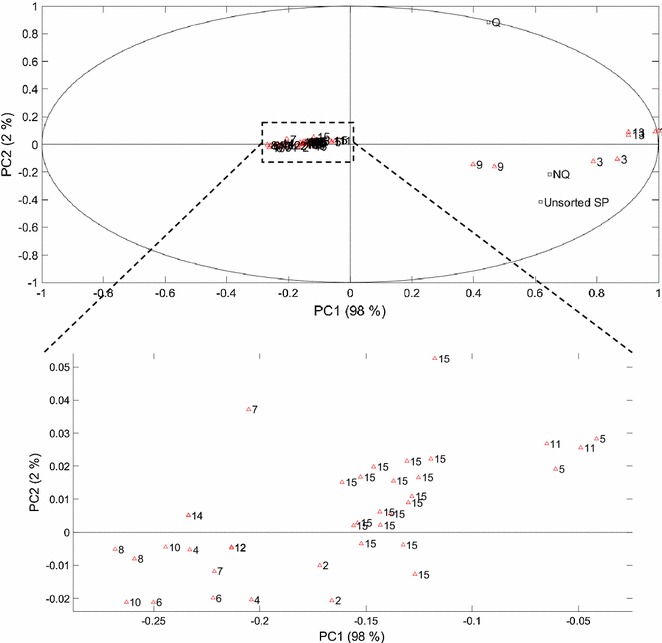
PCA analysis of factorial design experiment using unsorted SP-, Q- or NQ-cells as inoculum. Loading plot (*squares*) and Score plot (*triangles*)

A principal component analysis (PCA) of the three datasets combined was made to re-organize the information into new variables [principal components (PC)] that accounted for the majority of the variability in growth (Fig. 4). The input variables were set as yeast responses in terms of biomass formed after 14 h for the unsorted ESP-cells, Q-cells and NQ-cells. PC1 showed 98% variance with a high positive component loading for all subpopulations (Unsorted ESP-cells, 0.62; Q-cells, 0.44; NQ-cells, 0.65), demonstrating that growth of the three inocula was similar in the different media and was in agreement with the ANOVAs. A variation between the inocula was, however, seen in PC2 (2%), where Q-cells had a positive component loading (0.88), whereas the unsorted ESP-cells and NQ-cells were more similar to each other and had a slightly negative loading for biomass titre (unsorted ESP-cells, −0.42; NQ-cells, −0.22). From the score plot, it could be read that the largest influence of the different behaviour of Q-cells compared to ESP-cells and NQ-cells in both PC1 and PC2 was from growth in media M1, M3, M9 and M13, while the other media clustered together.

To further analyse potential differences between the three inocula, lag times, maximum growth rates and the final biomass in an intermediate inhibitory medium (3.5 g L^−1^ acetic acid, 1 g L^−1^ furfural and 1 g L^−1^ vanillin) were estimated by fitting the modified Gompertz equation (Eq. 1) [33] to the experimental data

1 $$y = A\exp \left\{{- \exp \left[{\frac{{\mu_{\max} \times e}}{A}\left({\lambda - 1} \right) + 1} \right]} \right\}$$

where *y* is the logarithm of the relative population size [ln(*N*/*N*_0_)], *A* is the asymptote [ln(*N*_∞_/*N*_0_)], *µ*_max_ is the maximum specific growth rate (h^−1^), *λ* is the lag time (h), *e* is exp(1) and *t* is time (h).

Modelling of growth responses for unsorted ESP-, Q- and NQ-cells revealed a small difference in lag time (18 and 7% longer for Q-cells than for unsorted ESP-cells and NQ-cells, respectively, *p* < 0.001) (Table 3; Additional file 2: Figure S1). The maximum specific growth rate and the asymptote, A, were similar for the different inocula, meaning that once growth started the specific condition rather than the history of the population determined the growth rate and biomass yield.

**Table 3:** Fitting of the Gompertz equation to experimental data obtained for cultivation in medium 15 (*n* = *18*)

Inoculum	Lag time *λ* (h)	Max. Growth rate *μ*_max_ (h^−1^)	Final population size *A* ln(OD/OD_0_)
Unsorted ESP-cells	10.6 ± 1.3	0.18 ± 0.01	1.9 ± 0.3
Q-cells	12.6 ± 0.8	0.17 ± 0.01	2.0 ± 0.2
NQ-cells	11.8 ± 0.3	0.18 ± 0.01	2.1 ± 0.1

**Fig. 5:**
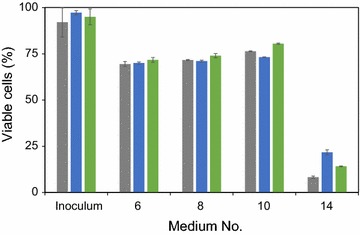
Cell viability after 24 h incubation in medium supplemented with different amounts of acetic acid, vanillin and furfural at pH 3.7. SP- cells (*grey*), Q-cells (*blue*) and NQ-cells (*green*). *Error bars* represent standard error of biological duplicates (*n* = 2)

Altogether the different analyses pointed towards that the shift into Q-cells was not determining the enhanced ability of ESP-cells to grow in the presence of inhibitors. In contrast, the trend was towards longer lag phases for Q-cells than for NQ-cells. On the other hand, since the lag time is determined by the number of viable cells at the start, it could also be that Q-cells were less tolerant to the inhibitors resulting in an initial drop in viability. However, FCM analysis after incubation for 24 h in static media demonstrated that cell viability was similar for unsorted ESP-cells, Q- and NQ-cells (Fig. 5). For medium M14 cell viability was substantially lower than for the other static media, demonstrating that this specific combination of inhibitors was most toxic.

### Intracellular pH distribution responses to lignocellulosic inhibitors at low external pH

Determining viability might not be an appropriate means to distinguish the ability to initiate growth in the presence of lignocellulosic inhibitors at low pH as viability of cells in static media remained also very high. Instead, the number of cells that are capable to maintain their pHi may be a better measure to predict the occurrence of growth when exposed to the harsh conditions. In a previous study, it was found that growth in the presence or absence of acetic acid at low extracellular pH correlated with the number of cells maintaining their pHi [38, 39]. To test this for ESP-cells, the response in pHi distribution in a CEN.PK strain over-expressing recombinant pHluorin was measured at lignocellulosic conditions by using ratiometric flow cytometry. Furthermore, measuring the response at the single-cell level could reveal any discrepancies in tolerance distribution within the different pre-culture populations.

The CEN.PK background was chosen because it is a well-established model strain for physiological studies within the yeast community [40], and it allowed for easy introduction of the pHluorin reporter system using the *URA3* marker. Further, it was previously established that induction of acetic acid tolerance at low pH is displayed both in TMB3500 and in the laboratory strain CEN.PK 113-7D [11], and is thus strain-independent.

**Fig. 6:**
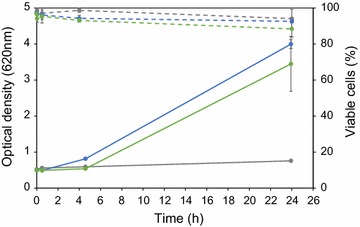
Effect of pre-cultivation on subsequent cultivation of yeast (CEN.PK 113-5D expressing pHluorin, TMB3800) in inhibitory medium. OD620 (*full lines*) and viability (*dotted lines*) as a function of cultivation time for the following inocula: LP-cells (*grey*), pre-adapted cells (*blue*) and ESP-cells (*green*). *Error bars* represent standard error of biological duplicates (*n* = 2)

**Fig. 7:**
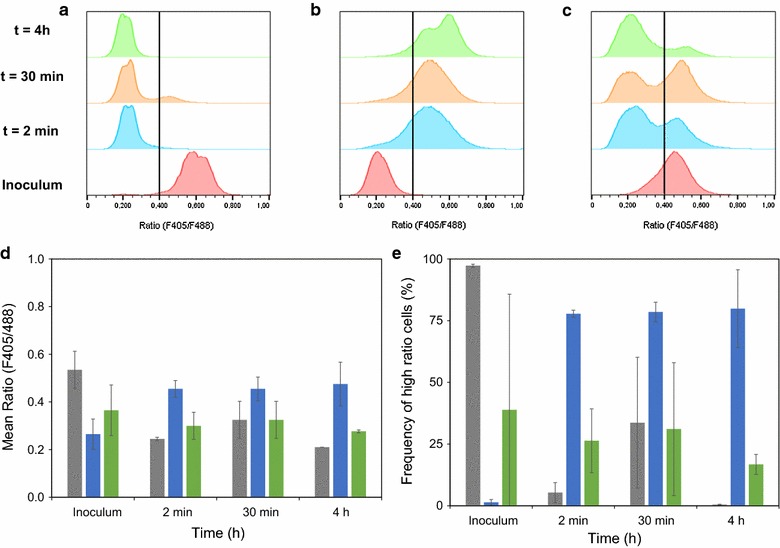
Evolution of pHi in different yeast cell inocula (CEN.PK 113-5D expressing pHluorin, TMB3800) upon exposure to a mixture of lignocellulosic inhibitors. Representative histograms from the following inocula: **a** LP-cells, **b** cells pre-adapted in medium supplemented with the inhibitors at pH 5, and **c** ESP-cells. The *vertical line* displays cells with high and low ratio (F405/F488) and was defined as the number (=0.400) separating LP-cells (**a**, inoculum) into fractions of 1% lowest and 99% highest percentiles. **d** Mean ratio (F405/F488) and **e** frequency of cells with high ratio (>0.400) for the three different inocula (LP-cells, *grey bars*; pre-adapted cells, *blue bars*; ESP-cells, *green bars*) measured over time. *Error bars* represent standard error of biological duplicates (*n* = 2). Ratio (F405/F488) is the ratio of pHluorin fluorescence emission collected at 542/50 and 533/30 nm originating from excitation at 405 and 488 nm, respectively. Ratiometric flow cytometry measurement was performed with a Moflo cell sorter and data analysis was performed with Flowjo

The CEN.PK strain was pre-cultured with or without acetic acid at pH 5.0, harvested in LP or in ESP and subsequently re-inoculated in defined mineral medium supplemented with 6 g L^−1^ acetic acid, 0.2 g L^−1^ vanillin and 0.75 g L^−1^ furfural at pH 4.5. Variation in pre-cultivation conditions indeed resulted in significant differences in growth profiles (Fig. 6, Additional file 3: Figure S2). LP-cells did not grow in the presence of inhibitors within the measured time interval of 24 h; it only grew in the control medium at pH 5.0 without supplementation of inhibitors (Additional file 4: Figure S3). Loss of viability could not explain the lack of growth for LP-cells since viability slightly reduced to about 85% during the first hour after which this level remained throughout the cultivation (Fig. 6). However, the number of LP-cells with kept physiological pHi had drastically reduced (Fig. 7a, e). Between the time of inoculation and before stable acquisition of cells in the FCM analysis (2 min), 95% of the population had a reduction in the fluorescence ratio from 0.54 to 0.25 (Fig. 7a, d, e). This demonstrates that nearly the total LP-cell population was sensitive to the inhibitory medium. Interestingly, a subpopulation (34 ± 27%) with a transiently higher ratio was observed after 30 min, but after 4 h the frequency of cells with high pHi was close to zero (Fig. 7a, d, e).

Cells from the pre-adapted inoculum had a lower initial pHi than LP-cells in the medium without inhibitors (Fig. 7b, d, e). As cells were transferred to the medium with inhibitors at pH 4.5, a majority of the population (78 ± 1%) recovered to a higher pH and maintained it under the measured time period (Fig. 7b, d, e). The more uniform pHi response to the shift in environment and the low fraction of cells with low pHi indicates that inhibitor tolerance was relatively homogenously distributed within the pre-adapted cell population.

Finally, cell populations in ESP cultures displayed a high degree of heterogeneity with a high frequency of cells with low pHi prior to inhibitor exposure (Fig. 7c–e). Upon transfer to the inhibitory medium, a distinct subpopulation had an increase in ratio to a similar level as those from the pre-adapted cells, while a majority of cells kept a low pHi over the 4-h incubation period. Despite this, cells with low pH were still viable as measured with FCM analysis of PI-stained cells (Fig. 6). Altogether, this indicates that the ability of ESP populations to initiate growth under lignocellulosic conditions is only present in a fraction of the cells.

### ROS level distribution responses to lignocellulosic inhibitors at low external pH

**Fig. 8:**
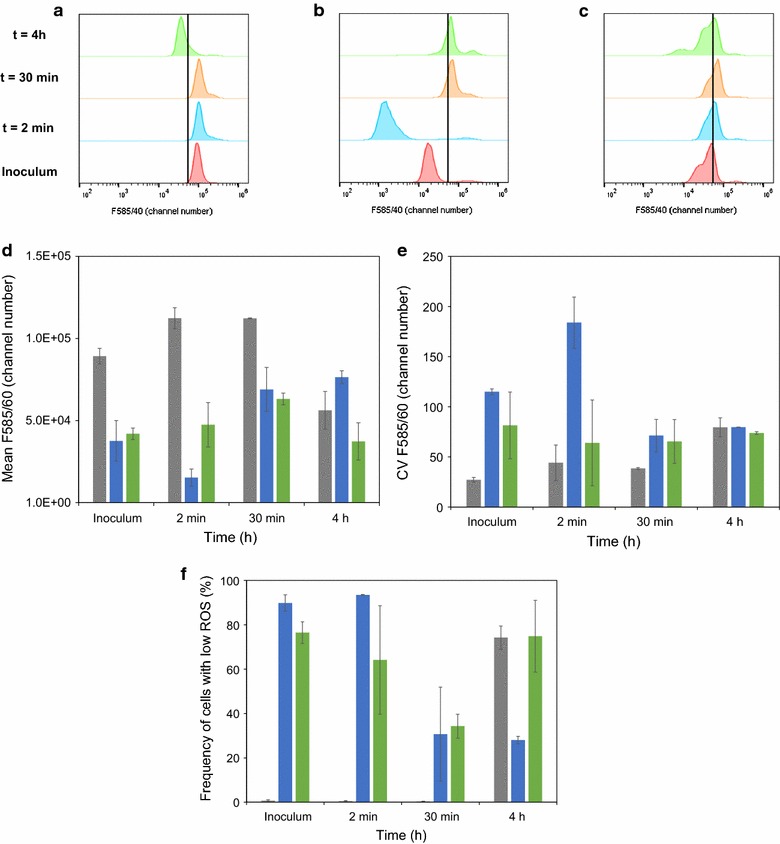
Evolution of ROS in different yeast cell inocula (CEN.PK 113-7D) upon exposure to a mixture of lignocellulosic inhibitors. Representative histograms from the following inocula: **a** LP-cells, **b** cells pre-adapted in medium supplemented with the inhibitors at pH 5 and **c** ESP-cells. The vertical line displays cells with high and low ROS levels and was defined as the number (F585/40 = 52 × 10^4^ channel number) separating the LP-cells (**a**, inoculum) into fractions of 1% lowest and 99% highest percentiles. **d** Mean F585/40 and **e** coefficient of variation (CV) of F585/40 for the three different inocula (LP-cells, *grey*; pre-adapted cells, *blue*; ESP-cells, *green*) measured over time. **f** Frequency of cells with low ROS levels over time. *Error bars* represent standard error of biological duplicates (*n* = 2). F585/40 is DHE fluorescence emission collected at 585/30 nm originating from excitation at 488 nm, respectively. Flow cytometry measurement was performed with a BD Accuri instrument and data analysis was performed with Flowjo. Histograms are from a representative experiment

Low ROS levels have previously been associated with cell tolerance to multiple stress factors, e.g. exposure to furfural [41] or oxidative stress from hydrogen peroxide [42]. Therefore, we hypothesized that the ability to quench ROS contributes favourably to an acquired inhibitor tolerance observed herein for pre-adapted and ESP-cells. To verify this, population responses to exposure to the mixture of lignocellulosic inhibitors at low pH were analysed by FCM. It was found that all cultures consisted of cells with high or low ROS levels (Fig. 8), although the subpopulation distribution differed considerably (Fig. 8a–c, f). Mean ROS levels were ca. twofold higher for LP-cells than for pre-adapted cells and ESP-cells (Fig. 8d). As cells were transferred to the inhibitory environment, a subpopulation of the pre-adapted inoculum had a dramatic reduction (ca 3-fold) in ROS levels already within 2 min, whereas a slight increase was observed for LP-cells. ESP-cells displayed a higher degree of heterogeneity (Fig. 8e) and had a large cell fraction with low ROS levels (Fig. 8c, f) that was more stable than for the other two inocula over the measured time period. After 4 h, the differences between the inocula were levelled out, i.e. ROS levels for LP-cells were significantly reduced, while they were slightly increased for the pre-adapted cells (Fig. 8d).

## Discussion

### ESP-cells displayed increased tolerance to lignocellulosic inhibitors at low pH

In this study, we demonstrated that early stationary phase cells have an increased tolerance to a combination of acetic acid, vanillin and furfural at low pH, as were reflected in immediate ability to initiate growth. The tolerance of ESP-cells was in range with the tolerance obtained by pre-adaptation in moderately inhibitory conditions. This was in stark contrast with exponentially growing cells that displayed a higher sensitivity towards all tested inhibitors. Allowing cells to enter ESP in the pre-cultivation step may therefore be beneficial from a process perspective as it will shorten the latency phase of the fermentation. It is well established from the previous studies that ESP-cells have high tolerance towards multiple stress factors [17, 19, 20]. Although the underlying mechanism behind tolerance is specific for each stress factor, cross-tolerance to different stressors is often observed and is generally ascribed to induction of the ESR upon nutrient depletion.

### Inhibitor tolerance was similar for Q- and NQ-cells in ESP populations

SP-cells were previously found to consist of two subpopulations with distinct physiological state, i.e. the so-called Q- and NQ-cells [24]. The cell fractions can easily be separated from each other based on differences in buoyant cell density (*δ*_Q_ = 1.14 g L^−1^ and *δ*_NQ_ = 1.10 g L^−1^), for example, by density gradient centrifugation [24] or by flotation as described in the present study. Q-cells consists of young mother cells and unbudded daughter cells and was previously shown to have high mitochondrial activity, low ROS levels, high levels of reserve carbohydrates (glycogen and trehalose), and a high ability to re-enter the cell cycle upon nutrient-rich conditions. NQ-cells, on the other hand, are more heterogeneous and consist of cells with genomic instability, high levels of ROS, non-functional mitochondria and display apoptotic characteristics. With regard to stress tolerance, Q-cells possess higher ability to withstand heat shock and carbon starvation than NQ-cells [24, 26]. We therefore reasoned that the higher ability of ESP-cells to initiate growth in the presence of lignocellulosic inhibitors at low pH was due to differentiation into Q-cells. However, the ability of Q-cells to proliferate and remain viable was rather similar to that of NQ-cells under the whole range of tested conditions in our study. It follows that differentiation into Q-cells was not the underlying cause for improved tolerance of ESP-cells. Engineering of Q/NQ distributions in the pre-cultivation step may therefore not be a way forward to minimize the lag phase of a lignocellulosic fermentation process. Q-cells actually had a slight prolongation of the lag phase upon inoculation to new medium, which may be due to that they are arrested in the G_0_ phase, and once nutrients are provided they require time for metabolic and structural rearrangements before entering the mitotic cell cycle. NQ-cells on the other hand are arrested in different phases of the cell cycle without clear preference for a specific phase, and did not require the same time before initiating proliferation. Furthermore, any negative effect of long-term starvation of NQ-cells, as observed previously [26], was not observed in ESP. It can be deduced from our results that cells in late SP would behave differently compared to ESP-cells.

### Vanillin was biocidal in combination with acetic acid at low pH

Yeast was sensitive to all tested inhibitors, although the degree of inhibition differed depending on the physiological state of the population. Acetic acid and vanillin were most detrimental for cell fitness, while furfural was inhibiting to a lesser extent at the concentration range used in the present study. The latter was probably due to an inherent high capacity of yeast to reduce furfural to the corresponding non-inhibitory alcohol, since pre-adapted cells behaved similarly to ESP-cells despite having a manifold higher specific furfural reductase activity (Additional file 5: Figure S3). It cannot be ruled out that the mechanisms behind the acquired inhibitor tolerance were different between pre-adapted cells and ESP-cells, and that furfural detoxification contributed favourably to the former. Inhibition was in most cases static rather than cidal, except for the combination of high amounts of vanillin (1.84 g L^−1^) and moderate levels of acetic acid (3.5 g L^−1^). At these concentrations, both vanillin and acetic acid were static on their own despite the low pH (3.7). The static effect of vanillin has been described previously [43], but herein we demonstrated that it is biocidal in combination with acetic acid at low pH. The underlying reasons for this is yet unknown, but it can be speculated that above a threshold concentration vanillin will inhibit carbon metabolism, resulting in an inability of the cell to efflux dissociated acetic acid and to keep pH homeostasis. Hence, the combination of vanillin and acetic acid has a synergistic effect on growth inhibition. Furthermore, our results indicated that furfural and acetic acid had a lower synergistic effect on growth inhibition. This is in contrast to a previous study on the combinatorial effect of different inhibitors (acetic acid, furfural and p-hydroxybenzaldehyde) in which it was found that furfural and acetic acid interacted synergistically on cell growth and ethanol production [44].

### Tolerant cultures displayed characteristic population responses in pHi and ROS

Process control of yeast fitness distributions during fermentation requires identification of correlations between critical process parameters and cell properties that can be rapidly monitored at the single-cell level, such as with flow cytometry [45, 46]. Here we looked into pHi and ROS formation as two cell properties that correlate with inhibitor stress tolerance in yeast.

#### FCM measurement of intracellular pH

ESP-cells consist of two subpopulations with differences in intracellular pH, and addition of glucose in non-inhibitory medium results in recovery of physiological pH for the entire population [37]. By measuring responses in intracellular pH distribution as cells were transferred from the pre-culture to the inhibitory medium, we observed that only a subpopulation of ESP-cells was able to maintain their pHi. This implicates that inhibitor tolerance of ESP cultures lies at the level of a subpopulation. A similar behaviour was previously observed for *Zygosaccharomyces bailii*, which was shown to have a 1000-fold higher fraction of cells in ESP that was tolerant to weak organic acids, e.g. benzoic acid, sorbic acid and acetic acid, than exponentially growing cells [18]. Tolerance to acetic acid has previously been found to correlate to the cell pHi prior to exposure of the stress [47]. In our case, the average pHi of the ESP-cells was lower than for LP-cells, which is in agreement with the previous studies [30, 37]. Pre-adapted cells also had a lower pHi than LP-cells and a majority of the population was able to recover physiological pH within 2 min. Low pHi may be beneficial due to a lower amount of dissociated acid in the cytoplasm at the moment the cells are exposed to the harsh lignocellulosic conditions. It is unclear how cells with initially low pHi are able to rapidly restore intracellular pH, as was the case for pre-adapted cells and a subpopulation of the ESP-cells (Fig. 7). Maintaining physiological pH is a requirement for a functional glycolysis [48], enabling ATP-driven proton export to keep pH homeostasis under acidic condition (see review [49]). Rapid reduction in free inorganic phosphate due to the formation of fructose bisphosphate may play a role in bringing the pHi to neutral as was monitored in *Lactococcus lactis* (see review by [50]). Yet, there might be other specific mechanisms contributing to the reduction of acetic acid stress in combination with vanillin and furfural.

#### FCM measurement of ROS levels

A correlation between inhibitor tolerance and a high frequency of cells with low ROS levels was observed. This may be due to an increased capacity of inhibitor tolerant populations to quench ROS that was formed spontaneously or as a consequence of inhibitor exposure [41]. ROS are formed continuously in mitochondria under aerobic conditions, and are generally at higher level in exponentially growing cells than in SP-cells [51]. This may explain the observed discrepancies in inhibitor tolerance that were observed between pre-adapted cells, LP-cells and ESP-cells. The lower ROS levels of pre-adapted cells may be caused by inhibitor-specific mechanisms, for example, by an increased ability to reduce furfural by an induced furfural reductase activity. Another contributing factor may be an increased level of glutathione, which scavenges ROS by non-enzymatic oxidation, and is involved in maintaining redox homeostasis in the cell via NADPH-dependent glutathione reductase (GLR1) [52]. It was previously shown that by increasing intracellular glutathione levels by over-expression, the gene coding for γ-glutamylcysteine synthetase (*GST1*) resulted in improved growth in non-detoxified spruce hydrolysate [53]. An alternative way to reduce ROS levels is to introduce a biosynthetic pathway to L-ascorbic acid (vitamin C) that functions as a scavenger for oxygen radicals [54]. Our results give support to reduction of ROS levels as a suitable target for improving tolerance to inhibitors.

## Conclusions

In this study, we demonstrate that cells in early stationary phase have increased tolerance to lignocellulosic inhibitors at low pH. Thus, allowing cells to enter ESP by carbon starvation during pre-cultivation may be a useful strategy to improve productivity in batch processes that are based on actively growing cells as biocatalysts for bioconversions that are limited by high amounts of inhibitors and low pH. Furthermore, flow cytometry as means to characterize population response profiles has demonstrated to be a sophisticated tool for prediction of yeast behaviour. Herein, we found that FCM-measured frequency of cells that recovered pHi and kept low ROS levels correlated with the ability of the yeast culture to initiate growth in harsh lignocellulosic conditions.

## Additional files

**Additional file 1.** ANOVA:s of factorial design experiment using unsorted ESP-, Q- or NQ-cells as inoculum.

**Additional file 2.** Fitting of the Gompertz equation to experimental data obtained for unsorted ESP-cells, Q-cells and NQ-cells.

**Additional file 3.** Viability and growth of CEN.PK 113-7D (w/o pHluorin) in defined medium supplemented with lignocellulosic inhibitors.

**Additional file 4.** Growth and fluorescence of CEN.PK 113-5D expressing pHluorin (TMB3800).

**Additional file 5.** Specific furaldehyde reductase activity in crude cell extracts.

## Abbreviations

LP: Log phase
ESP: Early stationary phase
SP: Stationary phase
Q: Quiescent
NQ: Non-quiescent
pHi: Intracellular pH
ROS: Reactive oxygen species
ESR: Environmental stress response
FCM: Flow cytometry
