# Supplementary material for: Increased lignocellulosic inhibitor tolerance of Saccharomyces cerevisiae cell populations in early stationary phase
Source: Biotechnol Biofuels. 2017 May 4;10:114. doi: 10.1186/s13068-017-0794-0 (PMC5418707; doi:10.1186/s13068-017-0794-0)
Supplement: Supplementary file 1 — Additional file 1. ANOVA:s of factorial design experiment using unsorted ESP-, Q- or NQ-cells as inoculum. [file 13068_2017_794_MOESM1_ESM.docx]

Additional file 1. ANOVA:s of factorial design experiment using unsorted ESP-, Q-, or NQ-cells as inoculum

Table S1 ANOVA with unsorted ESP-cells as inoculum

| **'Source'** | **'Sum Sq.'** | **'d.f.'** | **'Mean Sq.'** | **'F'** | **'Prob>F'** |
| --- | --- | --- | --- | --- | --- |
| 'aa' | 1,3 | 4 | 0,33 | 569,8 | 1,60e-28 |
| 'fur' | 0,040 | 3 | 0,01 | 20,10 | 2,00e-07 |
| 'van' | 2,0 | 3 | 0,68 | 1160,2 | 6,60e-32 |
| 'aa*fur' | 3,1e-05 | 1 | 3,1e-05 | 0,0523 | 0,821 |
| 'aa*van' | 0,62 | 1 | 0,62 | 1050,6 | 1,76e-25 |
| 'fur*van' | 8,6e-06 | 1 | 8,6e-06 | 0,0146 | 0,904 |
| 'aa*fur*van' | 0,0042 | 1 | 0,0042 | 7,0951 | 0,012 |
| 'Error' | 0,018 | 31 | 5,9e-04 |  |  |
| 'Total' | 4,0 | 45 |  |  |  |

Abbreviations: aa=acetic acid; fur=furfural; van=vanillin

Table S1 ANOVA with Q-cells as inoculum

| **'Source'** | **'Sum Sq.'** | **'d.f.'** | **'Mean Sq.'** | **'F'** | **'Prob>F'** |
| --- | --- | --- | --- | --- | --- |
| 'aa' | 0,45 | 4 | 0,11 | 261,3 | 2,13e-23 |
| 'fur' | 0,080 | 3 | 0,027 | 62,82 | 2,83e-13 |
| 'van' | 1,4 | 3 | 0,46 | 1062 | 2,52e-31 |
| 'aa*fur' | 0,013 | 1 | 0,013 | 30,18 | 5,21e-06 |
| 'aa*van' | 0,28 | 1 | 0,28 | 652,5 | 2,19e-22 |
| 'fur*van' | 0,042 | 1 | 0,042 | 98,51 | 3,83e-11 |
| 'aa*fur*van' | 0,0068 | 1 | 0,0068 | 15,70 | 4,05e-04 |
| 'Error' | 0,013 | 31 | 4,3e-04 |  |  |
| 'Total' | 2,3 | 45 |  |  |  |

Abbreviations: aa=acetic acid; fur=furfural; van=vanillin

Table S3 ANOVA with NQ-cells as inoculum

| **'Source'** | **'Sum Sq.'** | **'d.f.'** | **'Mean Sq.'** | **'F'** | **'Prob>F'** |
| --- | --- | --- | --- | --- | --- |
| 'aa' | 1,4 | 4 | 0,35 | 486,5 | 1,73e-27 |
| 'fur' | 0,034 | 3 | 0,011 | 15,54 | 2,38e-06 |
| 'van' | 2,3 | 3 | 0,76 | 1038 | 3,61e-31 |
| 'aa*fur' | 1,6e-05 | 1 | 1,6 | 0,02156 | 0,884 |
| 'aa*van' | 0,67 | 1 | 0,67 | 923,5 | 1,23e-24 |
| 'fur*van' | 0,0038 | 1 | 0,0038 | 5,242 | 0,0290 |
| 'aa*fur*van' | 0,00021 | 1 | 0,00021 | 0,2830 | 0,599 |
| 'Error' | 0,023 | 31 | 0,00073 |  |  |
| 'Total' | 4,4 | 45 |  |  |  |

Abbreviations: aa=acetic acid; fur=furfural; van=vanillin
