# Supplementary material for: Increased lignocellulosic inhibitor tolerance of Saccharomyces cerevisiae cell populations in early stationary phase
Source: Biotechnol Biofuels. 2017 May 4;10:114. doi: 10.1186/s13068-017-0794-0 (PMC5418707; doi:10.1186/s13068-017-0794-0)
Supplement: Supplementary file 2 — Additional file 2. Fitting of the Gompertz equation to experimental data obtained for unsorted ESP-cells, Q-cells and NQ-cells. [file 13068_2017_794_MOESM2_ESM.docx]

Additional file 2. Fitting of the Gompertz equation to experimental data obtained for Unsorted ESP-cells, Q-cells and NQ-cells

Figure S1. Fitting of the Gompertz equation to experimental data obtained for cultivation in medium 15 in microtiter plates (n=18). Fitting of the Gompertz equation was made for each replica with the solver function in Microsoft Excel to minimise the sum of least square, and the average model values were compared with all data points. a) Unsorted ESP-cells, b) Q-cells, and c) NQ-cells

a)

b)

c)
