# Supplementary material for: Increased lignocellulosic inhibitor tolerance of Saccharomyces cerevisiae cell populations in early stationary phase
Source: Biotechnol Biofuels. 2017 May 4;10:114. doi: 10.1186/s13068-017-0794-0 (PMC5418707; doi:10.1186/s13068-017-0794-0)
Supplement: Supplementary file 3 — Additional file 3. Viability and growth of CEN.PK 113-7D (w/o pHluorin) in defined medium supplemented with lignocellulosic inhibitors. [file 13068_2017_794_MOESM3_ESM.docx]

Additional file 4. Viability and growth of CEN.PK 113-7d (w/o pHluorin) in defined medium supplemented with lignocellulosic inhibitors

Figure S2 Viability (*dotted lines*) and growth (*full lines*) of CEN.PK 113-7d (w/o pHluorin) in defined medium supplemented with lignocellulosic inhibitors (6 g L-1 acetic acid, 0.2 g L^-1^ vanillin and 0.75 g L^-1^ furfural) at pH 4.5. Inocula: LP-cells (*orange*), pre-adapted cells (*yellow*), and ESP-cells (*green*).

Cells of the strain CEN.PK 113-7D from exponential phase and ESP grown in defined medium without inhibitors and pre-adapted cells from late exponential phase with inhibitors at pH 5 were transferred to a defined medium with inhibitors (6 g L^-1^ acetic acid, 0.2 g L^-1^ vanillin and 0.75 g L^-1^ furfural) at pH 4.5. Of these, the exponentially growing cells did not grow upon re-inoculation, but the pre-adapted and ESP cells displayed growth (Figure S2). The biomass followed the growth pattern with no increase in cell dry weight of exponentially growing cells after 24 h (0.38 ± 0.01 gdw L^-1^), whereas the pre-adapted and stationary cells grew after 24 h with a biomass of 1.27 ± 0.07 gdw L^-1^ and 1.12 ± 0.42 gdw L^-1^, respectively. After a rapid drop in viability from circa 95-100% to 80-90%, all the cultures had a high viability (more than 80%) over the remaining period of analysis (Figure S2). Altogether, this demonstrates that ESP cells indeed have an improved tolerance to the inhibitors compared to growing cells, and is in range with the one reached for pre-adapted cells.
