# Supplementary material for: Increased lignocellulosic inhibitor tolerance of Saccharomyces cerevisiae cell populations in early stationary phase
Source: Biotechnol Biofuels. 2017 May 4;10:114. doi: 10.1186/s13068-017-0794-0 (PMC5418707; doi:10.1186/s13068-017-0794-0)
Supplement: Supplementary file 4 — Additional file 4. Growth and fluorescence of CEN.PK 113-5D expressing pHluorin (TMB3800). [file 13068_2017_794_MOESM4_ESM.docx]

Additional file 3. Growth and fluorescence of CEN.PK 113-5D expressing pHluorin (TMB3800).

Figure S3 a) Growth and pHluorin fluorescence (ex 488nm/ em 533 nm) in control medium at pH 5.0 without supplementation of inhibitors using LP-cells (CEN.PK 113-5D expressing pHluorin, TMB3800) as inoculum. b) Average growth and standard error (*n*=4) during the first 7 hours. Cells were grown in shake flasks at 30C in a shake incubator set to 180 rpm, and growth was monitored by measuring optical density at 620 nm with a spectrophotometer. Single wavelength fluorescence intensity (ex 488/em 533 nm) of pHluorin was measured with a BD Accuri flow cytometer and data analysis was made in FlowJo, as described in the material and methods section of the paper.

a)

b)
