# Supplementary material for: Increased lignocellulosic inhibitor tolerance of Saccharomyces cerevisiae cell populations in early stationary phase
Source: Biotechnol Biofuels. 2017 May 4;10:114. doi: 10.1186/s13068-017-0794-0 (PMC5418707; doi:10.1186/s13068-017-0794-0)
Supplement: Supplementary file 5 — Additional file 5. Specific furaldehyde reductase activity in crude cell extracts. [file 13068_2017_794_MOESM5_ESM.docx]

Additional file 5. Specific furaldehyde reductase activity in crude cell extracts

Figure S4. Specific activity (mU mg^-1^) of furaldehyde reductase in crude cell extracts of CEN.PK 113-7D in LP-cells in defined mineral medium w/o inhibitors at pH 5.0, pre-adapted cells in defined medium supplemented with inhibitors (6 g L^-1^ acetic acid, 0.2 g L^-1^ vanillin and 0.75 g L^-1^ furfural) at pH 5.0 and ESP cells grown in defined mineral medium w/o inhibitors at pH 5.0.

## Enzymatic assay

Cell extracts were prepared using the Y-PER reagent (Pierce, Rockford, USA). Total protein concentration was determined using coomassie blue dye with bovine serum albumin (2 mg mL^-1^) as standard (ThermoFisher Scientific, Rockford, USA). Furfural reductase activity was measured as described in [48], using furfural (10 mM) as substrate, NADPH (200 μM) as cofactor and 5 μL of cell extract in potassium phosphate buffer (50 mM, pH 7). The oxidation of NADPH was followed by the change in absorbance at 340 nm using a multiscan ascent spectrophotometer (ThermoFisher Scientific, Sweden) at 30°C. The molar absorption coefficient (ε_340_) was 6.22 mM^-1^ cm^-1^ for NADPH. One unit of reductase activity corresponds to the formation of 1 μmol of NADP^+^ per minute.
